# Editorials

**Published:** 1897-10

**Authors:** 


### HOMŒOPATHIC PHYSICIAN,

A MONTHLY JOURNAL OF

HOMŒOPATHIC MATERIA MEDICA AND CLINICAL MEDICINE.

"If our school ever gives up the strict inductive method of Hahnemann, we are lost, and deserve only to be mentioned as a caricature in the history of medicine."—CONSTANTINE HERING.

Vol. XVII. OCTOBER, 1897. No. 10.

### EDITORIALS.

CALCAREA-CARB.—Calcarea is indicated in those having full habit, especially young people. The skin and muscles are lax.

Calcarea hemorrhages are bright red.

It sometimes develops diseases lying dormant in the system, causing them to, as it were, declare themselves, so that efficacious prescriptions may be given.

Calcarea has sleeplessness before midnight. There is much restlessness and tossing about the bed.

Calcarea has fever with much thirst. The fever is increased by drinking cold water.

Calcarea has a tertian type of fever. Its period of recurrence is in the evening, and there is first heat in the face and then the chill. The chill may come on after sleeping.

Calcarea has chilliness when rising in the morning. This is a special characteristic. The chilliness appears to be internal. The chill is worse in bed and after meals, and may be accompanied with clonic spasms. There is thirst during all the stages of the paroxysm, and drinking cold water, as before stated, aggravates the heat stage.

Calcarea has perspiration from the least exertion. This is a characteristic. The perspiration is less in-doors, and worse in the open cold air.

Natrum-carb. has perspiration after eating.

Silicea has perspiration in the morning.

The Calcarea perspiration occurs during sleep, especially before midnight, while under Silicea it comes after midnight.

The Calcarea perspiration is worse about the head, neck, and shoulders. Sometimes it is confined entirely to the front part of the body.

Calcarea is a very appropriate remedy for moist scurfy eruptions, such as "milk crust."

The pains of Calcarea are always worse in the morning.

The itching of eruptions abates by scratching under Calcarea.

The pains in the joints are always worse from the heat of the bed.

Calcarea is often useful for sore throat and stiff neck.

It has great sensitiveness to moist cold air. It is indicated in bad effects from Quinine, Phosphorus, Digitalis, Zinc, Sulphur, and Nitric-acid. It is a great remedy for children. It has desire to be magnetized like Phos.

It has aggravation from the loss of animal fluids. It is worse from cold weather, from lying on the left side, after sleeping too long, or from lying with the head high. It has aggravation from satisfying one's appetite.

It also has aggravation from suppressed perspiration. This is similar to Mercurius, Ipecac., and Nitric-acid.

Calcarea has amelioration from lying on the painful side, in dry weather, from lying on the back with the head low, and from lying on the right side.

Calcarea is an antidote to the effects of Bismuth.

Calcarea cannot be well repeated in adults, but in young children it *may* be repeated, and the younger the child the oftener may the dose be given.

Food Adulteration.—The United States Department of Agriculture is instituting an investigation into the extent of food adulteration. This is one of the most important subjects that can be brought before the medical profession. The department desires as much information as can be obtained. To this end it has issued a circular letter to all who are interested, asking for information. This circular, together with the letter accompanying it to the editor, is published in full on another page. It is hoped that some among our readers may have valuable information which they are willing to impart. This they are earnestly requested to do at their earliest opportunity. It should be borne in mind that only facts, not any theories, are wanted by the department.

